# Diagnostic Utility of Routine Serum Inflammatory Markers for Early Postoperative Infection, Hypoxia, and Hypotension After Fracture Surgery: A Prospective Cohort Study

**DOI:** 10.7759/cureus.111530

**Published:** 2026-06-26

**Authors:** Ravi Sharma, Mohamed Azlam P, Jayant Kothale, Vishal Kumar, Nitin Sahu

**Affiliations:** 1 Orthopaedics, Netaji Subhash Chandra Bose Medical College (NSCB) Medical College and Hospital, Jabalpur, IND; 2 Orthopaedics, Government Medical College, Seoni, Seoni, IND

**Keywords:** c-reactive protein, d-dimer levels, fracture-related infection, inflammatory biomarkers, post-fracture surgery

## Abstract

Introduction

Early detection of postoperative complications following fracture surgery remains challenging in resource-limited settings. Routine serum inflammatory markers, namely erythrocyte sedimentation rate (ESR), C-reactive protein (CRP), D-dimer, and white blood cell (WBC) count, are readily available, cost-effective biomarkers that may help identify patients at risk of early complications such as fracture-related infection (FRI), hypoxia, and hypotension.

Methods

In this prospective observational cohort, 282 adults (18-65 years) with acute fractures presenting within 24 hours of trauma underwent surgical fixation at a tertiary care center between December 2022 and June 2024. Serial ESR, CRP, D-dimer, and WBC measurements were obtained from admission through postoperative day 14. Patients were monitored for FRI, hypoxia requiring oxygen therapy, and hypotension requiring inotropic support. Associations between biomarker trajectories, prespecified quantitative thresholds, and subsequent complications were analyzed.

Results

Of 282 patients (225 males, 57 females; mean age 39.31 years), a total of 50 patients (17.7%) developed complications: 26 (9.2%) FRI, 16 (5.7%) hypoxia, and 21 (7.5%) hypotension. CRP exhibited the strongest association with FRI; failure of CRP to decline and levels ≥30 mg/L around postoperative day 5 were frequently observed before clinical signs of infection. Elevated D-dimer levels, particularly preoperative values ≥4.0 mg/L and early postoperative values ≥3.0 mg/L, were associated with increased risk of hypoxia and hypotension, identifying a higher‑risk subgroup for closer hemodynamic and respiratory monitoring. ESR and WBC provided supportive but less discriminative information.

Conclusion

Serial CRP and D-dimer measurements offer practical, non-invasive tools for early risk stratification of postoperative infection, hypoxia, and hypotension after fracture surgery. Incorporating these markers into surveillance pathways may facilitate earlier recognition of deterioration and timelier intervention, especially in resource-constrained settings.

## Introduction

Fractures impose a substantial global healthcare burden, with implications for acute management and long-term recovery. Despite surgical advances, postoperative complications - fracture-related infection (FRI), respiratory compromise, and hemodynamic instability - continue affecting outcomes and resource utilization [1]. Early identification of at-risk patients remains critical in orthopedic trauma management. Inflammatory markers indicate tissue injury, systemic inflammation, and infection. Erythrocyte sedimentation rate (ESR), C-reactive protein (CRP), D-dimer, and white blood cell (WBC) count are routinely measured, offering accessibility, rapid turnaround, and cost-effectiveness [2]. However, their utility for predicting early postoperative complications after fracture surgery lacks comprehensive prospective characterization in resource-limited settings.

ESR reflects red cell sedimentation influenced by acute-phase proteins, particularly fibrinogen. CRP, a hepatically-synthesized acute-phase protein, rises rapidly within 24-72 hours post-injury, with concentrations increasing from baseline (~0.8 mg/L) to >500 mg/L in severe trauma [3,4]. CRP demonstrates superiority over leukocyte counts and ESR in detecting surgical complications with bacterial etiology. D-dimer, a fibrin degradation product, is elevated in trauma, thromboembolism, infection, and acute respiratory distress. Recent evidence suggests D-dimer correlates with trauma severity and may serve as a prognostic indicator [5]. WBC count provides a readily available inflammatory marker, though its specificity for postoperative complications remains contested [6].

Fracture and surgical trauma trigger inflammatory cascades with characteristic temporal biomarker patterns. Understanding these patterns and their deviation in complications may enable early risk stratification and pre-emptive intervention. Prior studies examined individual markers in elective procedures or specific fractures, yet comprehensive prospective data evaluating multiple markers across diverse fracture locations for infection, hypoxia, and hypotension prediction remain limited [5-8].

The research gap was identified as the lack of prospective, serial biomarker data in fracture-surgery patients evaluating whether routine inflammatory markers can early identify distinct postoperative complications such as FRI, hypoxia, and hypotension. Existing evidence is largely limited to single-marker studies, severe trauma populations, or studies focused on thromboembolic outcomes rather than a broader spectrum of early postoperative complications. Our study addresses this gap by prospectively evaluating serial CRP, ESR, D-dimer, and WBC measurements at predefined peri-operative time points in a fracture‑surgery cohort from a resource‑limited tertiary center, directly relating biomarker trajectories and pragmatic cut-offs to early FRI, hypoxia, and hypotension. Hence, this study aims to evaluate the diagnostic accuracy of serial ESR, CRP, D-dimer, and WBC levels in predicting early postoperative complications after fracture surgery and to identify optimal biomarker thresholds for clinical risk stratification.

## Materials and methods

Study design and setting

This prospective observational, non-interventional study was conducted in the Department of Orthopaedics at Netaji Subhash Chandra Bose (NSCB) Medical College and Hospital, Jabalpur, Madhya Pradesh, India, a tertiary trauma referral center, between December 2022 and June 2024. The study protocol was approved by the Institutional Ethics Committee of NSCB Medical College (IEC/NSCBMC/22/8629-32), and written informed consent was obtained from all participants before enrollment.

Sample size and sampling strategy

The minimum required sample size was estimated using the formula for proportions in a single cohort: $n = \frac{(Z_{\alpha/2})^{2} \times p(1 - p)}{d^{2}}$, where Zα/2 is the standard normal deviate corresponding to a two-sided alpha of 0.05, p is the anticipated proportion of early postoperative complications after fracture surgery based on previous literature, and d is the desired absolute precision. Assuming an expected complication rate of approximately 20% from prior orthopedic trauma series and an absolute precision of 5%, the calculated sample size was 246 patients; to account for potential loss to follow-up and incomplete data, we aimed to recruit at least 280 participants. As planned, the minimum sample size was 280 participants, and 356 patients were assessed for eligibility; eligible patients were enrolled consecutively until the sample size of 282 was achieved.

Inclusion and exclusion criteria

We included adult patients aged 18-65 years presenting within 24 hours of acute traumatic fractures of long bones (femur, tibia-fibula, humerus, radius-ulna) or fractures involving the pelvis, clavicle, or vertebrae, who were scheduled for surgical fixation as definitive management. Patients were excluded if they declined consent or had chronic inflammatory or autoimmune disease, pre-existing cardiovascular disease, chronic liver or kidney disease, malignancy, or pregnancy. We also excluded individuals with isolated fractures of the hand or foot, polytrauma with significant associated blunt injury, pathological fractures, Gustilo-Anderson grade III open fractures, or traumatic amputations, to minimize baseline heterogeneity in systemic inflammatory and coagulation status.

Data collection and follow-up

At enrollment, demographic details, comorbidities, injury characteristics, American Society of Anesthesiologists (ASA) physical status classification, and relevant perioperative variables were recorded using a standardized case record form. Patients were followed prospectively from admission through postoperative day (POD) 14, with daily clinical assessment for signs of FRI, respiratory compromise, and hemodynamic instability. Any adverse events, reoperations, or intensive care admissions were documented. No proprietary scores or copyrighted scales beyond the ASA physical status classification, for which no special permission is required for descriptive clinical use, were employed in this study.

**Figure 1:**
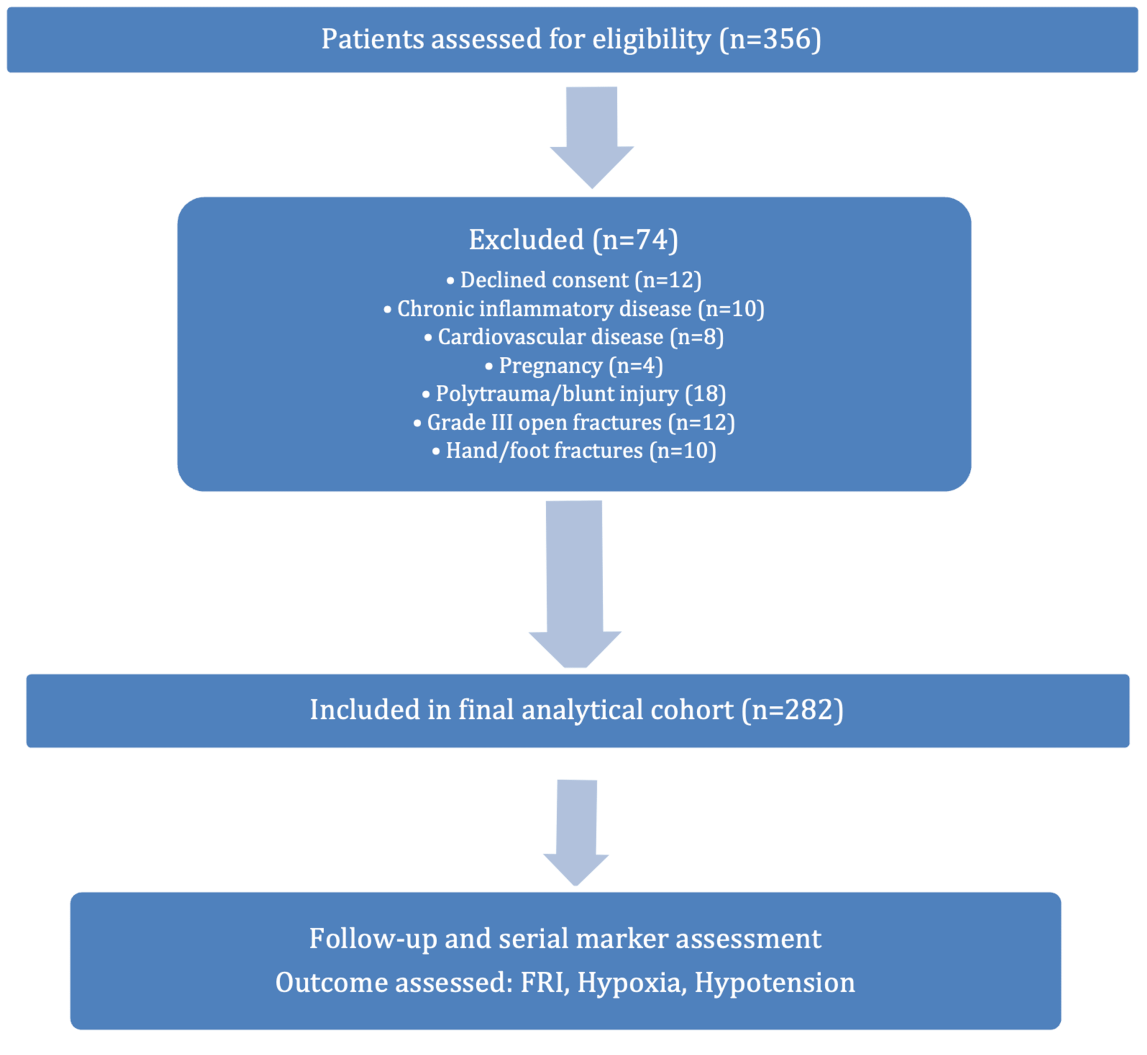
Flow diagram illustrating patient recruitment process Image created by the authors using Microsoft PowerPoint (Microsoft® Corp., Redmond, WA). FRI, fracture-related infection

Figure 1 illustrates the patient recruitment process.

Laboratory measurements

Venous blood samples were obtained at eight prespecified timepoints: admission (day 1 post-injury), day 3 post-trauma, the day before surgery (preoperative), and POD 1, 3, 5, 7, and 14. ESR was measured using the Westergren method with a reference value of ≤30 mm/hour. Serum CRP concentrations were determined by latex agglutination immunoassay (reference value <5 mg/L). Plasma D-dimer was measured by fluorescence immunoassay with a normal reference range of <0.5 mg/L. Total WBC count was analyzed using a fully automated hematology analyzer, with normal reference values between 4,500 and 11,000 cells/μL. The laboratory measurements were performed in the hospital central laboratory according to standard operating procedures, using routine automated analyzers and established reference ranges. Internal quality control procedures were followed for all assays, and samples were processed in the same laboratory to maintain consistency across time points.

Outcome definitions

FRI was defined in accordance with international consensus criteria as the presence of a sinus tract communicating with the fracture or implant, purulent discharge, wound breakdown exposing bone or implant, or growth of the same microorganism from at least two deep tissue or implant specimens [1,4]. Hypoxia was defined as an oxygen saturation (SaO₂) ≤95%, a partial pressure of arterial oxygen (PaO₂) <80 mmHg, or a PaO₂/fraction of inspired oxygen (FiO₂) ratio <300 mmHg requiring supplemental oxygen [9]. Hypotension was defined as a systolic blood pressure <90 mmHg or <80% of the patient’s baseline value, sustained for more than 10 minutes or requiring vasopressor or inotropic support.

Statistical analysis

Data were entered into a password-protected spreadsheet and analyzed using a trial version of IBM SPSS Statistics for Windows, Version 25.0 (IBM Corp., Armonk, NY). Continuous variables are reported as mean ± standard deviation (SD) or median with interquartile range, as appropriate, and categorical variables as frequencies and percentages. Independent-samples t-tests were used to compare mean marker levels between patients with and without each complication at corresponding timepoints, while chi-square tests were employed to evaluate associations between prespecified quantitative marker thresholds and the presence of complications. A two-sided p-value < 0.05 was considered statistically significant.

## Results

Demographics and fracture characteristics

A total of 282 patients were enrolled, including 225 males (79.8%) and 57 females (20.2%), with a mean age of 39.31 years. Fracture distribution was as follows: tibia-fibula 84 (29.8%), femur 76 (27.0%), radius-ulna 43 (15.2%), humerus 32 (11.3%), multiple bones 32 (11.3%), clavicle 7 (2.5%), pelvis 5 (1.8%), vertebrae 3 (1.1%). A total of 78 patients (27.7%) had open fractures, whereas 204 (72.3%) had closed fractures.

Complication incidence

Complications occurred in 50 patients (17.7%): infection in 26 (9.2%) patients - 20 superficial and six deep infections; hypoxia in 16 (5.7%); and hypotension in 21 (7.5%). Infection onset occurred predominantly during POD 7-14. Hypoxia and hypotension manifested earlier, with 75% hypoxia and 76% hypotension cases on POD 1.

Peak inflammatory marker levels varied significantly by fracture location. Femur fractures demonstrated the highest D-dimer elevation at 4.97 mg/L on POD 1, approximately four times baseline levels. Multiple bone fractures exhibited the highest ESR (45.50 mm/hour on POD 5) and CRP (45.82 mg/L on POD 3) peaks. Across all fracture types, D-dimer consistently peaked on POD 1, ESR peaked on POD 5, and CRP peaked on POD 3, reflecting characteristic temporal inflammatory responses to surgical trauma (Table 1).

**Table 1:** Distribution of peak inflammatory marker levels by fracture location (n = 282) Values are mean ± standard deviation (SD). D-dimer is reported in mg/L, erythrocyte sedimentation rate (ESR) in mm/hour, C-reactive protein (CRP) in mg/L, and white blood cell (WBC) count in 10³/μL. POD, postoperative day

Fracture location	D-dimer peak (mg/L)	ESR peak (mm/hour)	CRP peak (mg/L)	WBC peak (10³/μL)
Femur (n = 76)	4.97 (POD 1)	43.74 (POD 5)	31.80 (POD 3)	10.49 (POD 1)
Tibia-fibula (n = 84)	3.35 (POD 1)	42.71 (POD 5)	28.75 (POD 3)	10.95 (POD 5)
Humerus (n = 32)	4.17 (POD 1)	36.13 (POD 7)	25.04 (POD 3)	10.53 (POD 5)
Radius-ulna (n = 43)	0.75 (POD 1)	20.93 (POD 5)	20.15 (POD 3)	9.67 (POD 1)
Multiple bone (n = 32)	3.97 (POD 1)	45.50 (POD 5)	45.82 (POD 3)	11.45 (POD 1)

Mean inflammatory marker levels between patients with and without complications revealed distinct predictive patterns. CRP demonstrated statistically significant elevation on POD 5 (46.04 ± 23.05 mg/L vs. 15.50 ± 23.86 mg/L; p = 0.008), POD 7 (42.88 ± 21.25 mg/L vs. 10.14 ± 15.52 mg/L; p = 0.001), and POD 14 (36.08 ± 15.82 mg/L vs. 5.24 ± 12.83 mg/L; p = 0.001) in infected versus non-infected patients. ESR showed significant differences on POD 5, 7, and 14 for infection (all p < 0.05). Preoperative D-dimer was markedly elevated in patients developing hypoxia (3.38 ± 1.79 mg/L vs. 1.09 ± 1.31 mg/L; p = 0.001) and hypotension (3.04 ± 1.67 mg/L vs. 1.09 ± 1.31 mg/L; p = 0.001), while POD 3 D-dimer elevation specifically predicted hypoxia (p = 0.001). WBC count showed significance only for late infection detection on POD 7 and 14 (p < 0.012 and p < 0.004, respectively) (Table 2).

**Table 2:** Temporal profiles of inflammatory markers in patients with and without postoperative complications (n = 282) Values are mean ± standard deviation (SD). Between-group comparisons of marker levels were performed using independent-samples t-tests. CRP, C-reactive protein; ESR, erythrocyte sedimentation rate; FRI, fracture-related infection; POD, postoperative day; WBC, white blood cell

Marker	Time	Infected (mean ± SD)	Non-infected (mean ± SD)	p (infection)	Hypoxic (mean ± SD)	Non-hypoxic (mean ± SD)	p (hypoxia)	Hypotensive (mean ± SD)	Normotensive (mean ± SD)	p (hypotension)
CRP (mg/L)	POD 5	46.04 ± 23.05	15.50 ± 23.86	0.008	30.00 ± 20.00	17.73 ± 25.13	0.08	22.00 ± 26.00	18.46 ± 25.36	0.57
	POD 7	42.88 ± 21.25	10.14 ± 15.52	0.001	28.00 ± 18.00	13.02 ± 19.58	0.06	18.00 ± 22.00	13.63 ± 19.98	0.40
	POD 14	36.08 ± 15.82	5.24 ± 12.83	0.001	18.00 ± 12.00	8.15 ± 15.67	0.12	12.00 ± 16.00	8.78 ± 15.88	0.45
ESR (mm/hour)	POD 5	45.92 ± 14.76	36.58 ± 1.48	0.014	42.00 ± 14.00	37.32 ± 14.84	0.26	40.00 ± 15.00	37.52 ± 14.87	0.49
	POD 7	45.54 ± 13.42	30.85 ± 1.53	0.016	40.00 ± 12.00	32.10 ± 13.46	0.08	38.00 ± 14.00	32.28 ± 13.44	0.06
	POD 14	40.92 ± 10.55	24.10 ± 1.64	0.001	32.00 ± 10.00	25.31 ± 10.82	0.07	30.00 ± 11.00	25.49 ± 10.84	0.06
D-dimer (mg/L)	Preop	1.27 ± 1.64	1.09 ± 1.31	0.91	3.38 ± 1.79	1.09 ± 1.31	0.001	3.04 ± 1.67	1.09 ± 1.31	0.001
	POD 1	3.54 ± 2.63	3.23 ± 2.80	0.20	6.31 ± 2.70	3.23 ± 2.80	0.22	5.86 ± 2.66	3.23 ± 2.80	0.02
	POD 3	0.76 ± 1.60	0.94 ± 1.62	0.20	3.41 ± 1.69	0.94 ± 1.62	0.001	2.80 ± 1.80	0.96 ± 1.63	0.06
WBC (10³/μL)	POD 7	15.14 ± 3.09	8.08 ± 3.10	0.012	12.00 ± 3.20	8.87 ± 3.24	0.06	11.00 ± 3.50	9.05 ± 3.29	0.12
	POD 14	13.21 ± 2.65	7.73 ± 2.45	0.004	11.00 ± 2.80	8.31 ± 2.79	0.08	10.50 ± 3.00	8.41 ± 2.80	0.08

**Figure 2:**
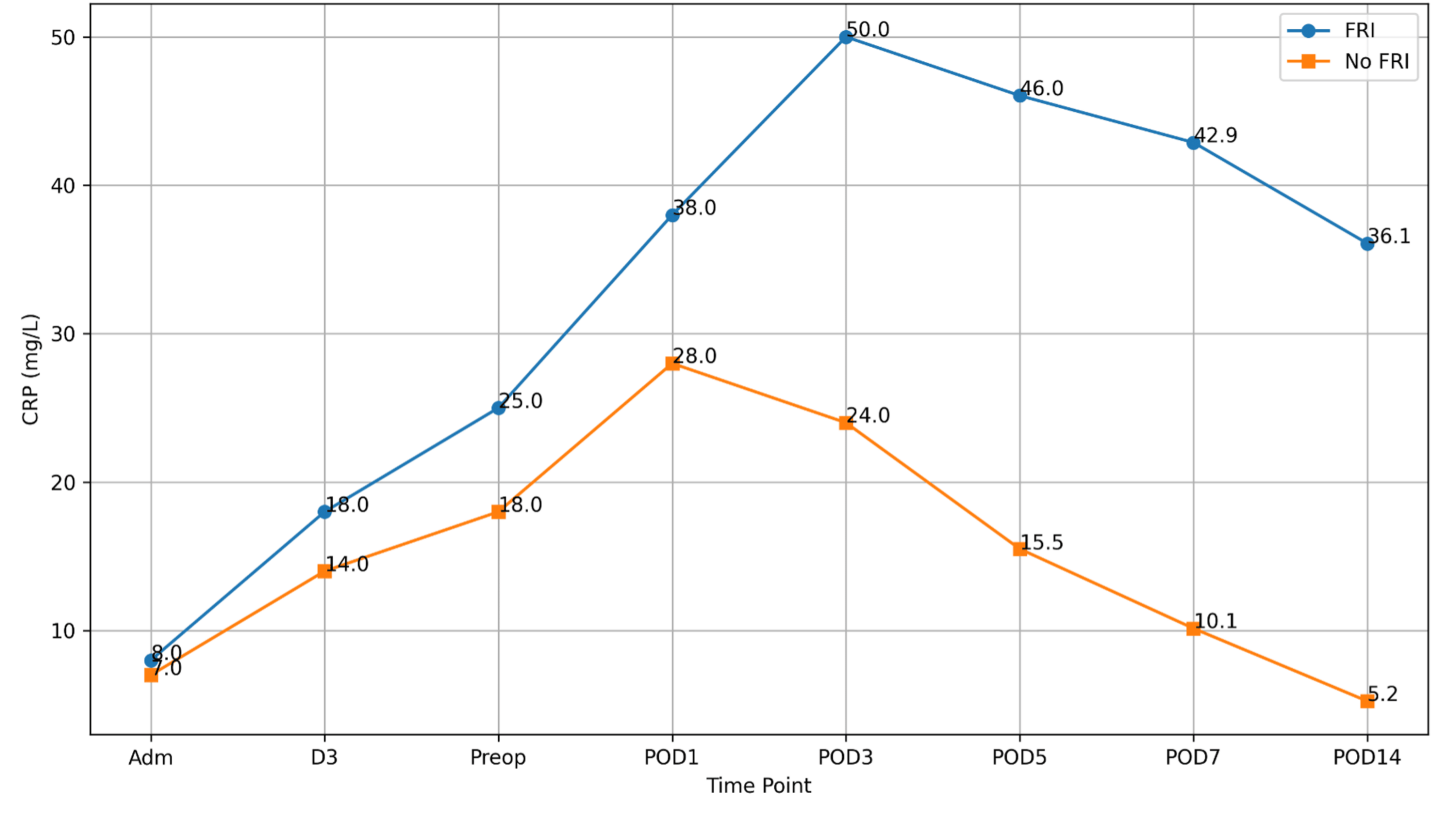
Mean C-reactive protein (CRP) levels in patients with and without fracture-related infection (FRI), with exact values shown at each time point Image generated by IBM SPSS Statistics for Windows, Version 25.0 (IBM Corp., Armonk, NY).

Figure 2 illustrates temporal trends in mean CRP levels from admission to POD 14 among patients with and without FRI.

**Figure 3:**
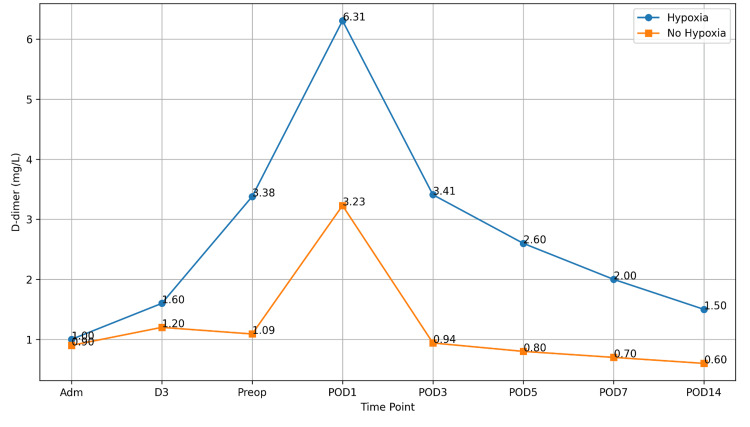
Mean D-dimer levels in patients with and without post-operative hypoxia, with exact values shown at each time point Image generated by IBM SPSS Statistics for Windows, Version 25.0 (IBM Corp., Armonk, NY).

Figure 3 illustrates temporal trends in mean D-dimer levels from admission to POD 14 among patients with and without postoperative hypoxia.

Quantitative threshold analysis enabled effective risk stratification. Preoperative D-dimer ≥4.0 mg/L (eight times the normal upper limit) identified patients with a 2.5-fold increased risk for hypoxia (27.8%) and hypotension (33.3%). POD 3 D-dimer ≥3.0 mg/L (six times normal) conferred a four-fold increased risk for both hypotension (45.5%) and hypoxia (36.4%). For infection prediction, POD 5 CRP ≥30 mg/L (six times normal) indicated a 2.4-fold increased risk (31.1%), while POD 7 CRP ≥50 mg/L (10 times normal) predicted a 3.4-fold increased risk (42.9%). These thresholds provide clinically actionable cutoffs for postoperative surveillance (Table 3).

**Table 3:** Risk stratification by quantitative inflammatory marker thresholds in postoperative fracture patients (n = 282) Threshold categories are defined as multiples of the upper limit of normal (N). Values are counts (n) with percentages in parentheses. Associations between marker thresholds and complications were assessed using chi-square tests. CRP, C-reactive protein; ESR, erythrocyte sedimentation rate; POD, postoperative day; WBC, white blood cell

Marker	Time	Threshold	n	Infection (%)	Hypoxia (%)	Hypotension (%)	No complication (%)
D-dimer	Preop	≥4.0 mg/L (8× N)	18	0 (0%)	5 (27.8%)	6 (33.3%)	10 (55.6%)
		≥2.0 mg/L (4× N)	54	3 (5.6%)	9 (16.7%)	9 (16.7%)	36 (66.7%)
	POD 1	≥4.0 mg/L (8× N)	108	11 (10.2%)	12 (11.1%)	12 (11.1%)	83 (76.9%)
		≥3.0 mg/L (6× N)	159	23 (14.5%)	15 (9.4%)	19 (11.9%)	119 (74.8%)
	POD 3	≥3.0 mg/L (6× N)	11	0 (0%)	4 (36.4%)	5 (45.5%)	6 (54.5%)
CRP	POD 3	≥30 mg/L (6× N)	79	14 (17.7%)	8 (10.1%)	10 (12.7%)	56 (70.9%)
	POD 5	≥30 mg/L (6× N)	45	14 (31.1%)	6 (13.3%)	7 (15.6%)	24 (53.3%)
	POD 7	≥50 mg/L (10× N)	14	6 (42.9%)	2 (14.3%)	2 (14.3%)	6 (42.9%)

## Discussion

This prospective study of 282 fracture patients systematically evaluated ESR, CRP, D-dimer, and WBC for predicting infection, hypoxia, and hypotension. CRP and D-dimer exhibited significant predictive value with specific temporal patterns enabling pre-symptomatic risk stratification.

CRP as infection predictor

Persistently elevated CRP on POD 5 predicted infection before clinical manifestation. Infected patients showed significantly higher CRP on POD 5, 7, 14 (p < 0.01), with mean POD 5 CRP 46.04 ± 23.05 mg/L versus 15.5 ± 23.86 mg/L in non-infected. Since clinical infection manifested predominantly after POD 7, elevated POD 5 CRP provided a two- to seven-day intervention window.

These findings align with prior studies. Metsemakers et al. [4] reported that CRP >96 mg/L after POD 4 demonstrated 92% sensitivity, 93% specificity for deep wound infection. Shetty et al. [5] documented CRP elevation before clinical symptoms in 7/9 infected patients. Ahmed et al. [6] demonstrated CRP correlation with infectious complications post-hip fracture surgery. 

CRP's characteristic trajectory in uncomplicated surgery - peaking POD 2-3 and progressively declining to normal by day 14-21 - is well-established [7,8]. Deviation (persistent elevation or secondary rise after day 5) reliably indicates infection. This reflects CRP's biology: rapid hepatic synthesis responding to IL-6 (24-72 hour rise), short half-life (18-20 hours), exponential decline upon inflammatory stimulus resolution [10,11].

D-dimer as hypoxia and hypotension predictor

D-dimer significantly predicted both complications. Preoperative D-dimer markedly elevated in patients developing hypoxia (3.38 ± 1.79 vs. 1.09 ± 1.31 mg/L; p = 0.001) and hypotension (3.04 ± 1.67 vs. 1.09 ± 1.31 mg/L; p = 0.001). Preoperative D-dimer ≥4.0 mg/L conferred 2.5-fold increased risk. POD 1 D-dimer ≥3.0 mg/L predicted hypoxia (p = 0.002), with 75% hypoxic patients exceeding this threshold.

D-dimer elevation reflects coagulation and fibrinolytic pathway activation, with magnitude correlating to injury severity [12,13]. Zhang et al. [14] demonstrated D-dimer correlation with fracture number and trauma severity. Our findings extend this by demonstrating predictive value for specific postoperative complications beyond thrombotic events.

The mechanistic link between elevated D-dimer and hypoxia or hypotension likely involves microvascular thrombosis, endothelial dysfunction, and systemic inflammatory response. Elevated D-dimer indicates ongoing fibrin formation and degradation, suggesting a prothrombotic state compromising pulmonary microcirculation (hypoxia) and systemic vascular resistance (hypotension) [15]. Ma et al. found reduced D-dimer clearance correlated with increased infection and thrombotic complications [16].

D-dimer showed limited infection prediction utility, contrasting with strong hypoxia and hypotension performance. This aligns with recent literature. Ebrahimpour et al. [17] reported no significant D-dimer difference between infected and non-infected orthopedic implant patients. Lu et al. [18] concluded D-dimer exhibited inferior sensitivity and specificity for periprosthetic joint infection versus CRP and ESR. While D-dimer reflects systemic inflammation and coagulopathy, it lacks bacterial infection specificity.

ESR patterns

POD 5, 7, and 14 ESR elevation significantly correlated with infection (p < 0.05), though delayed kinetics limit early detection. ESR peaked POD 5 in infected (45.92 ± 14.76 mm/hour) and non-infected (36.58 ± 1.48 mm/hour) patients, remaining persistently elevated in infected through day 14 (40.92 ± 10.55 vs. 24.1 ± 1.64 mm/hour; p = 0.001).

ESR's slow rise and prolonged elevation reflect dependence on acute-phase protein accumulation, particularly fibrinogen. Sharma et al. [19] and Krishna et al. [20] demonstrated ESR peaks five to seven days postoperatively, remaining elevated for weeks in uncomplicated cases, limiting diagnostic specificity. Our findings suggest ESR serves as a supportive marker combined with CRP rather than a standalone early predictor.

WBC count

WBC showed minimal predictive value except for late infection detection (POD 7, 14; p < 0.05). This aligns with prior studies. Paladino et al. [21] concluded that WBC cannot reliably differentiate major and minor trauma. Our data suggest WBC contributes limited incremental information beyond CRP and ESR.

FRI

In our cohort, 26 of 282 patients (9.2%) developed FRI, with 20 superficial and six deep infections. CRP showed the clearest association with infection, particularly on POD 5, and remained significantly higher on POD 7 and 14 in infected patients. This pattern is consistent with recent FRI literature showing that CRP, ESR, and leukocyte count have only limited standalone diagnostic accuracy and should be treated as suggestive rather than definitive markers in FRI diagnosis. Prior consensus work also emphasizes that FRI diagnosis requires integration of clinical findings, microbiology, imaging, and serum markers rather than reliance on inflammatory markers alone [22,23].

Clinical implications

The findings of this study support the potential role of serial inflammatory marker monitoring in postoperative surveillance for early recognition of complications. In particular, CRP measurement on POD 1, 3, and 5 may be useful, and a CRP value above 46 mg/L on day 5 or failure of CRP to decline after day 3 may indicate a higher risk of infection and justify closer clinical observation and further evaluation in selected high-risk patients. Similarly, preoperative D-dimer levels of ≥4.0 mg/L and POD 1 D-dimer levels of ≥3.0 mg/L may identify patients at increased risk of postoperative hypoxia and hemodynamic instability, supporting closer respiratory and hemodynamic monitoring. Overall, serial trends in CRP, D-dimer, and ESR appear more informative than any single marker alone, but the proposed thresholds should be interpreted as preliminary and require external validation before being adopted into routine clinical protocols.

Future directions include multicenter validation of the proposed CRP and D-dimer thresholds, integration of these markers into risk prediction models that incorporate clinical and imaging variables, and exploration of adjunctive biomarkers such as interleukin-6 or procalcitonin to further refine early detection of postoperative complications. Interventional studies evaluating protocolized postoperative surveillance and early targeted interventions in biomarker-defined high-risk patients may clarify whether this strategy translates into reduced morbidity, shorter hospital stay, and improved functional outcomes.

Strengths and limitations

Strengths of this study include its prospective design, relatively large sample, standardized serial sampling, and evaluation of multiple routinely available biomarkers in a clinically relevant fracture-surgery cohort from a resource-limited setting. However, the single-center design may limit generalizability, residual selection bias cannot be completely excluded, and the 14-day follow-up may have missed late deep FRIs. Additional limitations include the absence of molecular inflammatory mediators, the lack of external validation of proposed thresholds, and sample size estimation based on complication frequency rather than formal diagnostic-accuracy outcomes. As one of the methodological limitations, the study was primarily powered to estimate complication prevalence and biomarker associations rather than to provide definitive diagnostic-accuracy estimates for all markers and time points.

## Conclusions

This prospective study suggests that routine serum inflammatory markers, particularly CRP and D-dimer, may have useful diagnostic value for early postoperative risk stratification after fracture surgery. Persistent CRP elevation around POD 5 was associated with subsequent FRI, while elevated preoperative and early postoperative D-dimer levels were associated with hypoxia and hypotension. ESR appeared to provide supportive information for later infection trends, whereas WBC showed limited additional utility. The temporal patterns and candidate thresholds identified in this study may assist postoperative surveillance in similar settings; however, these findings should be interpreted cautiously and require external validation before routine clinical implementation.
